# Structure of angiogenin dimer bound to double-stranded RNA

**DOI:** 10.1107/S2053230X22008317

**Published:** 2022-08-30

**Authors:** Katharina Sievers, Ralf Ficner

**Affiliations:** a Department for Molecular Structural Biology, Georg-August-Universität Göttingen, Justus-von-Liebig Weg 11, 37077 Göttingen, Germany; Sungkyunkwan University School of Medicine, Republic of Korea

**Keywords:** angiogenin, ribonucleases, RNP, RNase A, double-stranded RNA

## Abstract

Angiogenin is an unusual member of the RNase A family and is of great interest in multiple pathological contexts. Although it has been assigned various regulatory roles, its core catalytic function is that of an RNA endonuclease. However, its catalytic efficiency is comparatively low and this has been linked to a unique C-terminal helix which partially blocks its RNA-binding site. Assuming that binding to its RNA substrate could trigger a conformational rearrangement, much speculation has arisen on the topic of the interaction of angiogenin with RNA. To date, no structural data on angiogenin–RNA interactions have been available. Here, the structure of angiogenin bound to a double-stranded RNA duplex is reported. The RNA does not reach the active site of angiogenin and no structural arrangement of the C-terminal domain is observed. However, angiogenin forms a previously unobserved crystallographic dimer that makes several backbone interactions with the major and minor grooves of the RNA double helix.

## Abstract 2

Angiogenin is a pathologically relevant but little understood ribonuclease, the interactions of which with RNA are structurally unknown. Here, the first crystal structure of human angiogenin bound to RNA is presented.

## 1. Introduction

Angiogenin, also referred to as RNase 5, is a small 123-amino-acid protein and a member of the bovine pancreatic ribo­nuclease A (RNase A) superfamily. Its common name refers to it first being identified as a factor that induces blood-vessel formation, a process known as angiogenesis (Fett *et al.*, 1985 ▸). Since then, angiogenin has been identified as a molecule of interest in a number of physiological processes and pathologies, most noticeably tumorigenesis (Tsuji *et al.*, 2005 ▸) and the neurodegenerative diseases amyotrophic lateral sclerosis (ALS) and Parkinson’s disease (reviewed by Prehn & Jirström, 2020 ▸).

Angiogenin from humans shares a high degree of sequence similarity with the eponymous member of the superfamily, and the catalytic triad consisting of a lysine (Lys40) and two histidines (His13 and His114) is conserved between the two enzymes (Supplementary Fig. S1). The protein exhibits the typical RNase A-like overall fold, although it lacks a fourth disulfide bond and instead features a cell receptor-binding site encompassing residues 58–70 (Hallahan *et al.*, 1991 ▸). Like RNase A, angiogenin has a tripartite binding pocket with sub­pockets to hold the scissile phosphate bond (P_1_) and the two bases located at the 5′ and 3′ ends of the cleavage site (B_1_ and B_2_) (Acharya *et al.*, 1995 ▸). However, angiogenin features a unique C-terminal 3_10_-helix and its B_2_ site is occluded by Gln117, which is locked in place through a hydrogen bond to Thr44. This occlusion is thought to be the reason for the remarkably reduced catalytic activity compared with RNase A that is typical of angiogenin proteins (Russo *et al.*, 1994 ▸). In addition to its lower activity, angiogenin is also much more substrate specific than RNase A. While likewise cleaving the 3′ side of pyrimidine nucleotides, it seems to do so only in the structural context of a few specific RNAs. Reported substrates are several rRNAs, tRNA and a type of promotor-associated RNA referred to as pRNA (Shapiro & Vallee, 1987 ▸; Rybak & Vallee, 1988 ▸; Lee & Vallee, 1989 ▸; Hoang & Raines, 2017 ▸). For this reason, it has long been suspected that the cleavage activity of angiogenin is improved through a rearrangement of the B_2_ site that only occurs upon the binding of physiological substrates or binding partners. A similar case has been demonstrated using structural data for salmon RNAse 2, although here the autoinhibitory effect is due to an insertion and not to the C-terminal region (Sica *et al.*, 2021 ▸; Supplementary Fig. S2). No structural data for angiogenin in an ‘activated state’ are available.

Angiogenin is a secreted protein and is translated with a 24-amino-acid N-terminal signal peptide which is cleaved during maturation (Kurachi *et al.*, 1985 ▸). The protein is reported to bind to receptors on the surface of endothelial and neuronal cells in an autocrine or paracrine manner, followed by endocytosis (Ferguson & Subramanian, 2018 ▸). In the cytosol, angiogenin is bound by Rnh1 through a remarkably tight interaction (with a *K*_d_ in the femtomolar range) and thus is kept in an inactive state (Lee *et al.*, 1989 ▸; Hoang & Raines, 2017 ▸). Angiogenin also contains a nuclear localization sequence (_30_MRRRG_35_) and promotes the transcription of rRNA (Moroianu & Riordan, 1994 ▸; Tsuji *et al.*, 2005 ▸). While it was initially thought to achieve this by binding to rDNA through an ‘ABE’ motif, recent evidence points to a mechanism in which angiogenin acts by cleaving a 97 nt promotor-associated RNA, which leads to the alleviation of rDNA silencing (Xu *et al.*, 2002 ▸, 2003 ▸; Hoang & Raines, 2017 ▸).

Another field of research on angiogenin with growing interest is based on the observation that angiogenin can, like some other RNases, cleave tRNAs within their anticodon loop, thereby generating tRNA halves, also referred to as tRFs or tiRNAs (Yamasaki *et al.*, 2009 ▸; Fu *et al.*, 2009 ▸; Su *et al.*, 2019 ▸). This is thought to occur in the context of stress granules in the cytoplasm and to be part of the cellular stress response, although this relationship is currently under debate (Emara *et al.*, 2010 ▸; Pizzo *et al.*, 2013 ▸; Sanadgol *et al.*, 2022 ▸). A large number of publications regarding tRNA fragments have emerged in recent years, and have recently been reviewed by Su *et al.* (2020 ▸), Polacek & Ivanov (2020 ▸) and Magee & Rigoutsos (2020 ▸).

An interesting phenomenon occurs in multiple members of the RNase A family, in which stable dimers with catalytic activity can be formed through a process of 3D domain swapping (Bennett *et al.*, 1995 ▸; Libonati & Gotte, 2004 ▸; Gotte *et al.*, 2012 ▸, 2021 ▸). Several modes of association exist, with either N- or C-terminal domains being exchanged between subunits, and sometimes even both. Notably, such RNase dimers cannot be bound and inhibited by Rnh1 (Murthy & Sirdeshmukh, 1992 ▸). Most recently, dimerization through N-terminal domain swapping has also been reported for angiogenin (Fasoli *et al.*, 2021 ▸).

Here, we present the crystal structure of two angiogenin molecules bound to an RNA duplex in a dimer-like fashion. This complex structure was obtained as a byproduct in an attempt to crystallize angiogenin with a tRNA anticodon stem loop, and while it is unlikely to have a direct equivalent *in vivo*, it does provide one example of the RNA-binding capabilities of angiogenin, for which no structural evidence exists so far.

## 2. Materials and methods

### 2.1. Production of the angiogenin (H114A)–RNA complex

A synthetic gene encoding mature human angiogenin (hAng), without its N-terminal signal peptide and codon-optimized for expression in *Escherichia coli*, was cloned into the pET-26b(+) plasmid (BioCat GmbH, Heidelberg, Germany). From this plasmid, an H114A mutant was generated by site-directed mutagenesis (BioCat GmbH, Heidelberg, Germany). For protein expression, *E. coli* BL21 (DE3) cells were transformed and grown in Terrific Broth medium with kanamycin (50 mg l^−1^) at 37°C. Once the culture had reached an OD_600_ of 0.8, isopropyl β-d-1-thiogalactopyranoside (IPTG) was added to a final concentration of 1 m*M*. Induced cultures were incubated for 3 h at 37°C with agitation and then harvested by centrifugation. The cells were washed once in 1× PBS, flash-frozen and stored at −20°C until further use. For purification, thawed cells from a 1 l culture volume were resuspended in lysis buffer (50 m*M* Tris–HCl pH 8.0, 2 m*M* EDTA) and ruptured using a microfluidizer unit (M-110S Microfluidizer; Microfluidics, Westwood, Massachusetts, USA). hAng was prepared from inclusion bodies and refolded as described previously (Holloway *et al.*, 2001 ▸). Briefly, the lysate was sonified and then centrifuged for 20 min at 20 000*g*. The pellet fraction was then washed once with lysis buffer containing 5%(*v*/*v*) Triton X-100 and once with only buffer, and was sonified and centrifuged in each step. Inclusion bodies were solubilized in 10 ml solubilization buffer (7 *M* guanidine–HCl, 0.15 *M* reduced glutathione, 2 m*M* EDTA pH 8.0, 0.1 *M* Tris–HCl pH 8.0) by gently stirring for 4 h or until fully dissolved. Refolding was achieved by dropwise dilution into 500 ml 0.5 *M* l-arginine pH 8.0, 0.6 m*M* oxidized glutathione. In preparation for ion-exchange purification, the solution was filtered (0.45 µm), diluted fourfold with distilled H_2_O and applied onto a 10 ml SP Sepharose FF column equilibrated in 25 m*M* Tris–HCl pH 8.0, 0.2 *M* NaCl. The target protein was eluted in a gradient to 1.1 *M* NaCl in 50 ml. Unlike as described previously, fractions containing the target protein were pooled, concentrated using a 3 kDa molecular-weight cutoff Amicon device (Merck, Darmstadt, Germany) and further purified by size-exclusion chromatography using a Superdex 75 16/600 column (Cytiva, Marlborough, Massachusetts, USA) in 20 m*M* Tris pH 8.0, 120 m*M* NaCl. The pure protein was concentrated to 9 mg ml^−1^ and stored at 4°C for several days until use.

N19-RNA with the sequence 5′-GCCCGCCUGUCACGCGGGC-3′ was synthesized by Axolabs (Kulmbach, Germany). The RNA was heated to 95°C for 5 min and annealed by snap-cooling on ice. hAng H114A was slowly mixed with a 1.2-fold molar excess of annealed RNA in 20 m*M* Tris pH 8.0, 120 m*M* NaCl to a final concentration of 5 mg ml^−1^ protein and was incubated on ice for 30 min.

### 2.2. Crystallization

Crystals that diffracted well were obtained from high-throughput screening. Freshly prepared complex was mixed with screening condition in a 1:1 or 2:1 ratio in a 3 Lens 96-well sitting-drop vapour-diffusion plate (SWISSCI, High Wycombe, United Kingdom) using a Mosquito pipetting robot (SPT Labtech, Melbourn, United Kingdom). Sealed plates were incubated at 20°C for ten days and then placed at 4°C for eight days. Crystallization information is summarized in Table 1 ▸.

### 2.3. Data collection and processing

Synchrotron X-ray diffraction data were collected on beamline P14 operated by EMBL Hamburg at the PETRA III storage ring, DESY, Hamburg, Germany. Diffraction images were indexed, integrated and scaled using the *XDS* package (Kabsch, 2010 ▸). Data-collection and processing statistics are summarized in Table 2 ▸.

### 2.4. Structure solution and refinement

The structure of the hAng (H114A)–RNA complex was solved by molecular replacement with *Phaser* (McCoy *et al.*, 2007 ▸) using the high-resolution structure of human angiogenin (PDB entry 5eop; Chatzileontiadou *et al.*, 2016 ▸) and nucleotides 26–44 of a tRNA model (PDB entry 6ugg; Chan *et al.*, 2020 ▸) remodelled as a polyuridine chain. After initial refinement with *REFMAC*5 (Murshudov *et al.*, 2011 ▸), the RNA model was completely rebuilt to fit the density of the RNA duplex. The structure was then iteratively refined using *REFMAC*5 with manual adjustment in *Coot* (Emsley *et al.*, 2010 ▸). In the final stages of refinement, *ERRASER* and *Phenix* were used (Chou *et al.*, 2016 ▸; Liebschner *et al.*, 2019 ▸). Refinement statistics are summarized in Table 3 ▸.

### 2.5. Analysis of surface electrostatics

Surface electrostatics were calculated using the Adaptive Poisson–Boltzman method (*APBS*) as implemented in *PyMOL* (Jurrus *et al.*, 2018 ▸).

## 3. Results and discussion

### 3.1. Crystallization and structure determination of human angiogenin H114A in complex with RNA

A complex of the inactive angiogenin variant H114A and a 19-mer RNA was prepared by mixing and incubation on ice. Previous experiments with RNA had shown that mixing angiogenin H114A with various types of RNA immediately resulted in precipitation, which was reversible when subsequently incubated for several hours. Precipitation was also observed to be temperature-dependent and formed more strongly at 4°C, while it was most easily reversed by equilibrating to room temperature.

Assuming that temperature-labile oligomers, in which several angiogenin molecules bound, and thereby bridged, more than one RNA molecule, were the cause of the previously observed precipitation, crystallization plates were placed at 4°C for further incubation when no crystals had appeared after ten days of incubation at 20°C. This yielded well diffracting pyramidal and bipyramidal crystals from which scattering data of sufficient quality could be collected.

Attempts at molecular replacement using an angiogenin search model alone were unsuccessful. Only upon including an RNA model could a solution be found. Although the RNA sequence was designed to form a stem-loop structure and a search model representing the anticodon stem loop from a tRNA model (PDB entry 6ugg) was used in molecular replacement, the density that emerged after initial refinement clearly showed a double-stranded RNA duplex structure. After the RNA model had been rebuilt as a duplex, *R*_free_ improved substantially. Placing water and buffer molecules further improved the models and after further refinement *R*_work_ and *R*_free_ finally converged to 0.205 and 0.268, respectively.

### 3.2. Structure overview and observed angiogenin–RNA interactions

**Figure 1:**
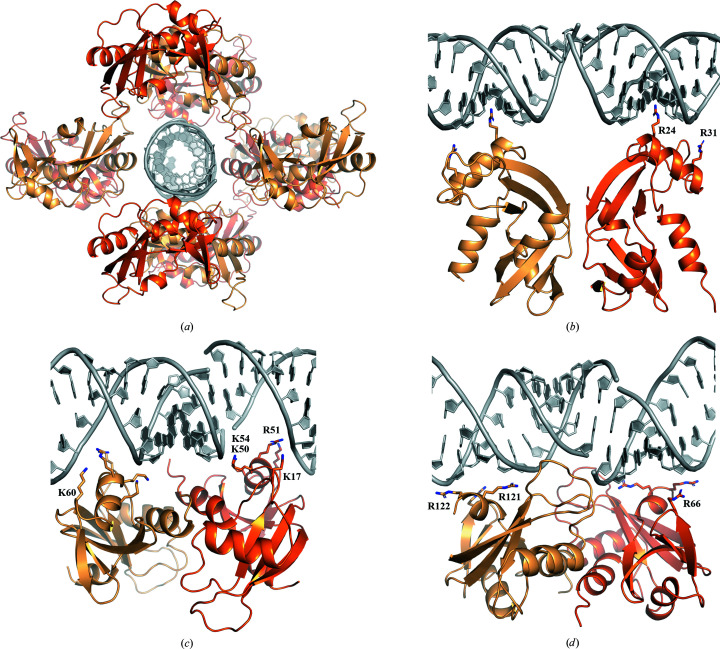
Angiogenin–RNA crystal lattice and structural overview. (*a*) Overview of the crystal lattice showing angiogenin chains along the RNA helix. (*b*) Contacts between the RNA duplex and angiogenin chains *A* and *B* via helices 2. (*c*) Major-groove contacts between the RNA duplex and angiogenin chains *A* and *B* via helices 3. (*d*) Minor-groove contacts between the RNA duplex and angiogenin chains *A* and *B* via the N-terminal helix.

The unit cell contains two molecules of angiogenin (chains *A* and *B*) and one double-stranded RNA duplex (chains *C* and *D*). Since the RNA sequence was not designed to be self-complementary, the RNA complex, made up of two strands of identical sequence, contains three mismatches. However, all of them are pyrimidine–pyrimidine pairings which do not cause steric clashes. In both copies, the space left open between the Watson–Crick edges of the paired nucleotides C32 and C38 is bridged by the guanidium group of Arg24 of chain *A* and *B*, which forms hydrogen bonds to the keto groups of both cytidines. The single U35/U35 pairing is not bridged by an amino acid, but local distortion of the backbone geometry allows a stabilizing hydrogen bond between O4 (U35 in chain *D*) and H3 (U35 of chain *C*). The 3′- and 5′-terminal bases stack against the 5′- and 3′-terminal bases of the next unit cell, thereby forming an extended RNA helix that spans the whole crystal. Considering all symmetry-related copies of the two angiogenin chains, the extended RNA helix is fully covered by associated protein (Fig. 1 ▸ *a*).

Each chain *A*/chain *B* angiogenin pair interacts with the RNA duplex via three different contact sites; each interaction is dominated by the contribution of one of the four helices in angiogenin (a secondary-structure overview is shown in Supplementary Fig. S1). All of these interactions are sequence-independent backbone contacts. For this reason, it is possible for the same interaction motif of the protein to bind to two different sections of the RNA duplex.

**Figure 2:**
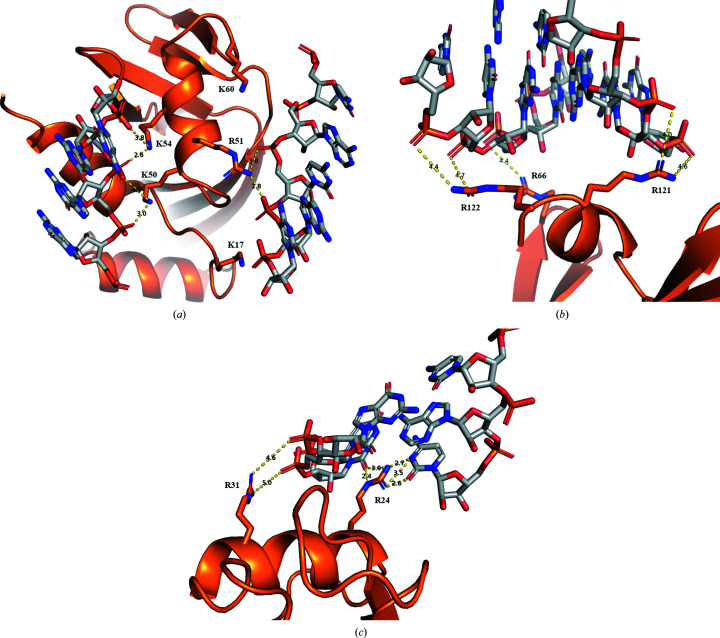
Electrostatic contacts between angiogenin and the RNA duplex. Hydrogen bonds and electrostatic interactions up to 5 Å are shown as dashed lines. The interatomic distance (Å) for each contact is given. (*a*) Contacts between the RNA duplex and angiogenin helix 3. (*b*) Contacts between the RNA duplex and angiogenin helix 4. (*c*) Contacts between the RNA duplex and angiogenin helix 2.

**Figure 3:**
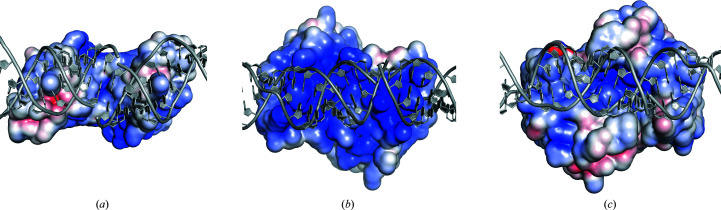
Surface electrostatics of RNA–angiogenin contact sites. (*a*) Surface electrostatics at the RNA contact site characterized by angiogenin helix 2. (*b*) Surface electrostatics of the crystallographic angiogenin dimer at the RNA contact site characterized by angiogenin helix 3. (*c*) Surface electrostatics of the crystallographic angiogenin dimer at the RNA contact site characterized by angiogenin helix 4.

The only exception is the aforementioned Arg24, which specifically bridges the space between C38 and C32 (Fig. 2 ▸ *c*). Another arginine, Arg31, binds to a backbone phosphate in the vicinity (Figs. 1 ▸ *b* and 2 ▸ *c*). Both arginines are part of helix 2. Protein–nucleic acid contacts are typically dominated by electrostatic forces. Angiogenin is a strongly cationic protein with a theoretical pI of 9.7 (Fig. 3 ▸) and helix 2 contains three adjacent arginines (Arg31-Arg32-Arg33) that are part of the nuclear localization sequence of angiogenin. Interestingly, the same residues were also involved in binding heparin in a previously published angiogenin structure (PDB entry 4qfj; Yeo *et al.*, 2014 ▸).

The most extensive contacts are formed by helix 3, which reaches deep into the major groove of the RNA helix and contributes Lys50, Arg51 and Lys54 to the interaction site (Figs. 1 ▸ *c* and 2 ▸ *a*). Lys17 and Lys60, while not part of helix 3, are located in the vicinity, although their distances to the closest backbone phosphates are greater than 5 Å. The arrangement of chains *A* and *B* is of optimal size to also occupy the major groove exactly one helix turn away, and the same residues are involved in contacts in both chains. The surface electrostatics of this contact reveal a large area of continuous, strong positive charge (Fig. 3 ▸ *b*). This makes a strong interaction with the RNA backbone unsurprising, even though this represents the face located opposite to the active sites.

Another RNA contact is mainly mediated by the angiogenin-specific C-terminal helix 4, which is located close to the active site. In both angiogenin chains this helix contributes Arg121 and Arg122, which interact with opposite strands across the minor groove (Figs. 1 ▸ *d* and 2 ▸ *b*). The interaction is further strengthened through the nearby Arg66 also binding to the backbone. Interestingly, the strongest positive charge on this contact face is located in a central pocket which contains the active sites of both proteins (Fig. 3 ▸ *c*).

However, the RNA duplex does not reach into the active site (Supplementary Fig. S3). The C-terminal helix which mediates the closest RNA contact at the same time acts as a gatekeeper and is positioned between the active site and the RNA. This helix is suspected to undergo a conformational change when binding to a physiological substrate to make the active site accessible. Here, the helix remains in an identical position in both chains (Supplementary Fig. S4) and the RNA binding that is observed in this structure does not trigger a structural rearrangement. Although the angiogenin molecules make extensive contacts with the RNA duplex, the structure is unlikely to resemble the arrangement during a cleavage event of a physiological substrate.

### 3.3. Potential dimerization sites

**Figure 4:**
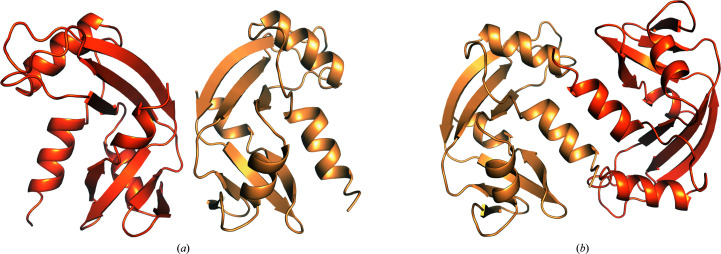
Angiogenin crystallographic dimers. (*a*) The crystallographic dimer sharing interface 1 is associated by the edges of the respective β-sheets 2 and 5. (*b*) The crystallographic dimer sharing interface 2 is held together by interaction of the N-terminal helices.

The observed crystal lattice has a fairly low solvent content of 41.61%. Neighbouring protein chains make very close contacts, which leads to the question whether they can provide insight into potential oligomerization sites in solution. The two contact types with the largest surfaces are symmetric and are mediated by the edges of β-sheets 2 and 5 of the protein and by its N-terminal α1 helices (Table 4 ▸). Although interface type 1 (Fig. 4 ▸ *a*) has a larger surface area, its solvation free-energy term (Δ^i^ *G*) is positive, with a Δ^i^ *G* *P*-value above 0.5, which is indicative of a hydrophilic interaction and an artefact of crystal packing (Table 4 ▸). In contrast, interface 2 (Fig. 4 ▸ *b*) is slightly smaller but has a negative solvation free-energy term and a *P*-value indicative of a largely hydrophobic interaction that is further stabilized by eight hydrogen bonds and four salt bridges.

While angiogenin is considered to be a primarily monomeric protein, the RNase A family as a whole is known to engage in various homodimeric configurations, including 3D domain-swapped arrangements. Recently, the first evidence of such a 3D-swapped dimer was reported for angiogenin (Fasoli *et al.*, 2021 ▸). Evidence was presented for an interaction via the N-terminal helix of angiogenin, but the accompanying homology model shows a different arrangement to the N-terminally mediated interface 2 that is described here. In addition, the density of the RNA-bound structure is unambiguous and clearly shows that no domains are exchanged between adjacent proteins. The angiogenin crystal structure with PDB entry 1b1j (Leonidas *et al.*, 1999 ▸) is the only structure to feature a similarly arranged crystallographic dimer. However, its chains are angled differently, and the two crystallographic dimers differ by a large r.m.s.d. value of 6.345 Å (Supplementary Fig. S5).

## 4. Conclusion

The interactions of angiogenin are manifold, are crucial to its function *in vivo* and are barely beginning to be understood. Even though the presented angiogenin–RNA complex does not occur *in vivo*, the observed RNA interactions might serve to derive the first structural models of more relevant interactions of angiogenin with known *in vivo* targets.

## Supplementary Material

PDB reference: human angiogenin bound to RNA duplex, 8af0

Supplemerntary Figures. DOI: 10.1107/S2053230X22008317/ek5029sup1.pdf

## Figures and Tables

**Table 1:** Crystallization

Method	Sitting-drop vapour diffusion
Plate type	3 Lens 96-well sitting-drop plate
Temperature (K)	277
Protein concentration in complex (mg ml^−1^)	5
RNA:protein ratio in complex	1.2:1
Buffer composition of complex solution	20 m*M* Tris pH 8.0, 120 m*M* NaCl
Composition of reservoir solution	200 m*M* NaCl, 20%(*w*/*v*) PEG 6000, 100 m*M* HEPES pH 7.0
Volume and ratio of drop	0.375 µl, 2:1 complex:reservoir ratio
Volume of reservoir (µl)	40

**Table 2:** Data collection and processing Values in parentheses are for the outer shell.

Diffraction source	DESY beamline P14
Wavelength (Å)	0.976250
Temperature (K)	100
Detector	Dectris EIGER2 CdTe 16M
Crystal-to-detector distance (mm)	374.26
Rotation range per image (°)	0.1
Total rotation range (°)	450
Exposure time per image (s)	0.01
Space group	*P*2_1_2_1_2_1_
*a*, *b*, *c* (Å)	50.38, 66.66, 101.40
α, β, γ (°)	90, 90, 90
Mosaicity (°)	0.184
Resolution range (Å)	45.12–2.43
Total No. of reflections	218261
No. of unique reflections	24805
Completeness (%)	99.8 (99.8)
Multiplicity	8.8 (7.8)
CC_1/2_	99.9 (69.4)
〈*I*/σ(*I*)〉	13.03 (1.49)†
*R*_meas_ (%)	0.0944 (1.663)
Overall *B* factor from Wilson plot (Å^2^)	70.47

† The cutoff was chosen according to CC_1/2_. The mean *I*/σ(*I*) falls below 2.0 at 2.53 Å resolution.

**Table 3:** Structure solution and refinement Values in parentheses are for the outer shell.

Resolution range (Å)	45.12–2.43 (2.62–2.43)
Completeness (%)	99.2
σ Cutoff	*F* > 1.35σ(*F*)
No. of reflections, working set	12625 (2467)
No. of reflections, test set	663 (132)
Final *R*_cryst_	0.205 (0.3330)
Final *R*_free_	0.268 (0.3800)
No. of non-H atoms	
Protein	1967
RNA	800
Ligand	24
Water	60
R.m.s. deviations	
Bond lengths (Å)	0.010
Angles (°)	1.443
Average *B* factors (Å^2^)	
Protein	83.49
RNA	84.87
Ramachandran plot	
Most favoured (%)	94.63
Allowed (%)	5.37
Outliers (%)	0.00

**Table 4:** *PISA* (Krissinel & Henrick, 2007 ▸) analysis of interfaces

Interface	Mediating elements	Interface area (Å^2^)	Δ^i^ *G* (kcal mol^−1^)	Δ^i^ *G* *P*-value	Hydrogen bonds	Salt bridges
1	β2/β2, β5/β5	650.0	1.0	0.611	10	0
2	α1/α1	534.1	−2.1	0.350	8	4
