# Supplementary material for: Structure of angiogenin dimer bound to double-stranded RNA
Source: Acta Crystallogr F Struct Biol Commun. 2022 Aug 30;78(Pt 9):330–7. doi: 10.1107/S2053230X22008317 (PMC9435672; doi:10.1107/S2053230X22008317)
Supplement: Supplementary file 1 [file f-78-00330-sup1.pdf]

STRUCTURAL BIOLOGY COMMUNICATIONS

Volume 78 (2022)

Supporting information for article:

Structure of angiogenin dimer bound to double-stranded RNA

Katharina Sievers and Ralf Ficner

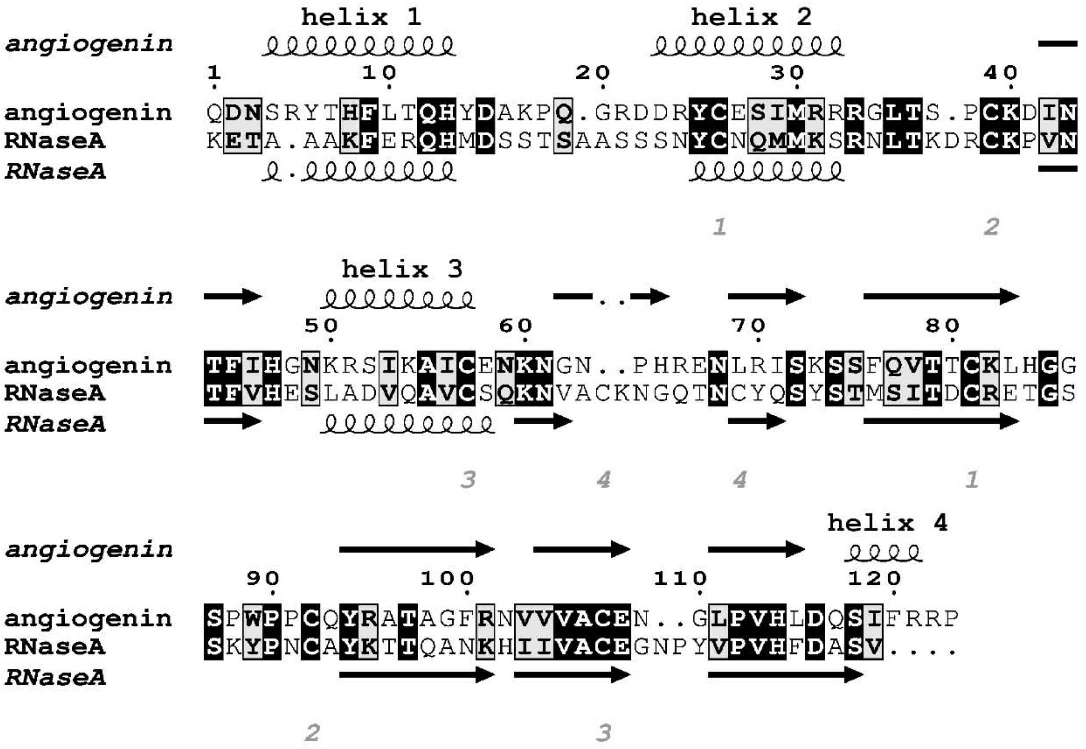

Figure S1 Multiple sequence alignment of human angiogenin and bovine pancreatic RNase A

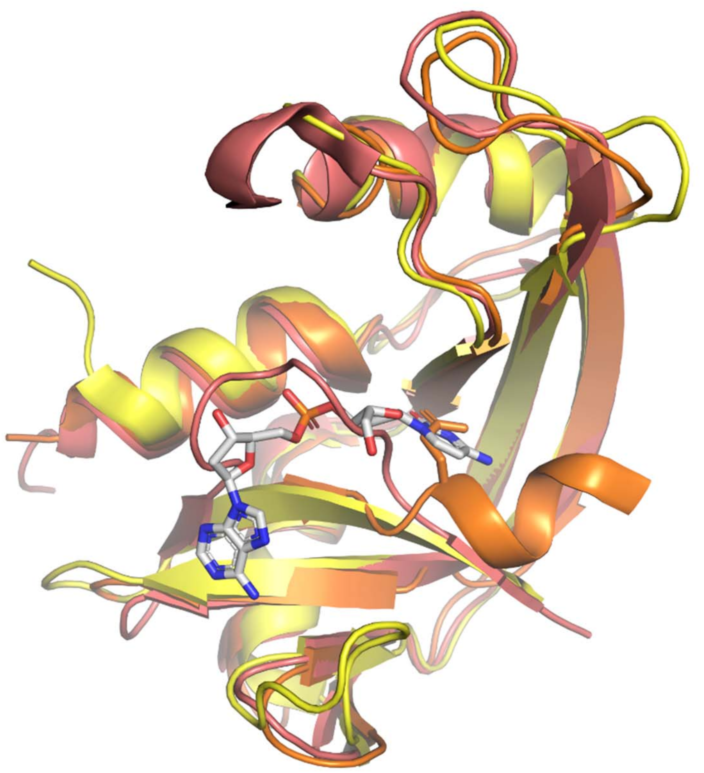

Figure S2 Structural comparison of bovine pancreatic RNase A, including a CpA dinucleotide bound to its active site (yellow, PDB-ID: 1TQ9), salmon RNase 2 (dark salmon, PDB-ID: 7BFK) and human angiogenin (orange, PDB-ID: 5EOP)

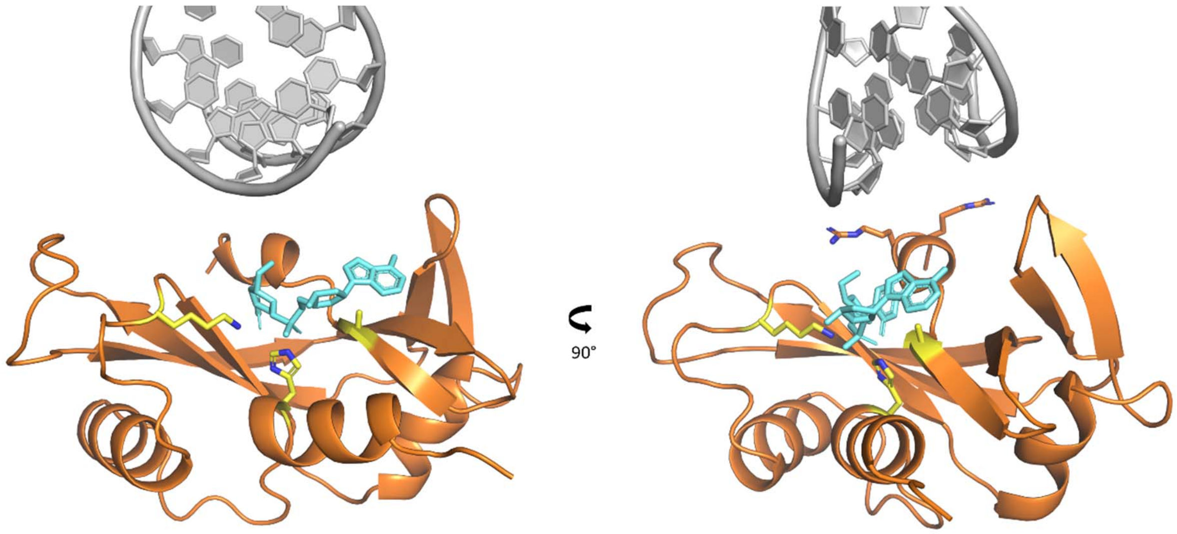

Figure S3 Chain A active site with nearest RNA. Catalytic residues H13, K40 and H114A are shown in yellow stick representation. A CpA dinucleotide bound in a published RNase A structure (PDB-ID: 1TQ9) is superimposed in cyan and marks the location of the active site binding pocket.

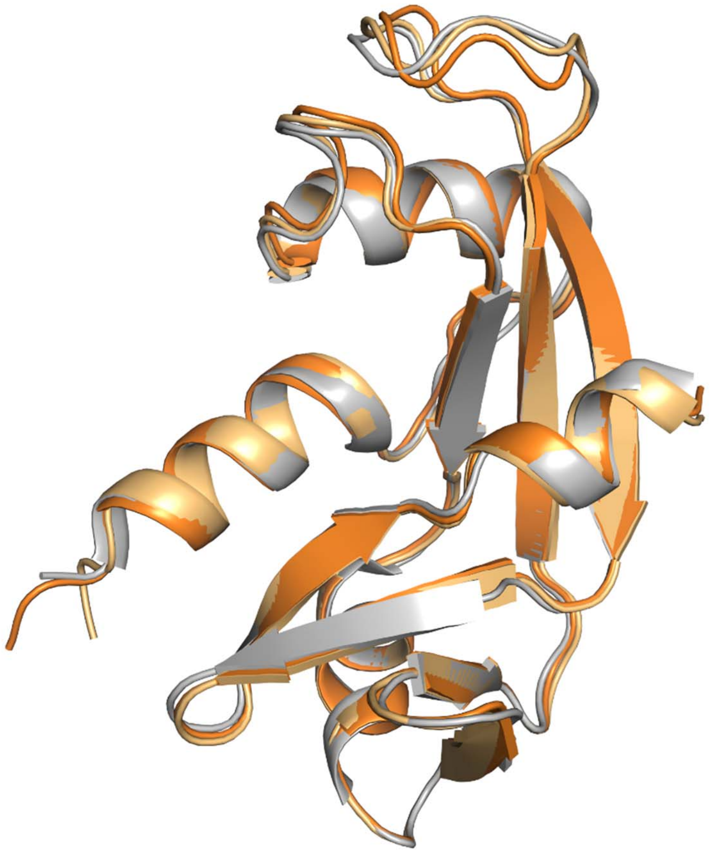

Figure S4 Overlay of chain A (dark orange), chain B (light orange) and angiogenin apo structure (grey, PDB-ID: 5EOP)

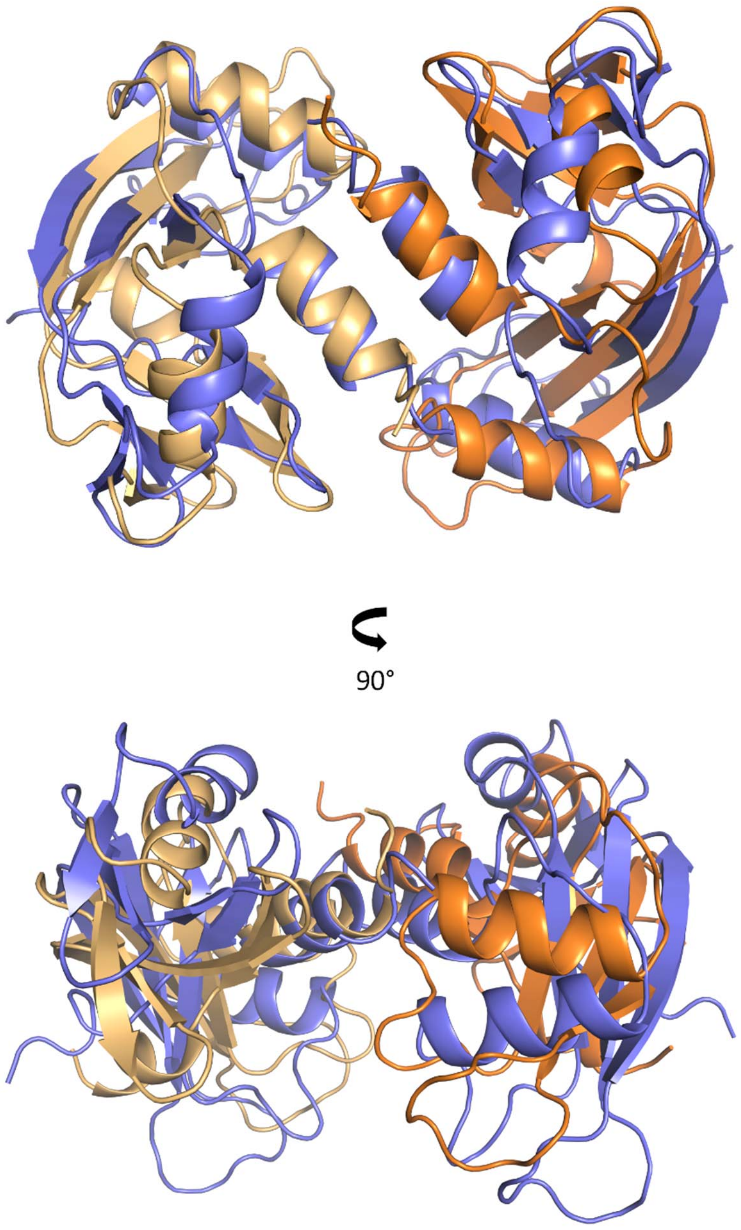

Figure S5 Comparison of reported crystallographic dimer (orange) with crystallographic dimer in previous angiogenin structure (blue, PDB-ID: 1B1J)
